# Vitamin D_3_ Protects against Diabetic Retinopathy by Inhibiting High-Glucose-Induced Activation of the ROS/TXNIP/NLRP3 Inflammasome Pathway

**DOI:** 10.1155/2018/8193523

**Published:** 2018-02-22

**Authors:** Li Lu, Qianyi Lu, Wei Chen, Jingwen Li, Chunxia Li, Zhi Zheng

**Affiliations:** ^1^Department of Ophthalmology, Anhui Provincial Hospital The First Affiliated Hospital of University of Science and Technology of China, Hefei 230001, China; ^2^Department of Ophthalmology, The First Affiliated Hospital of Soochow University, Suzhou 215006, China; ^3^Department of Ophthalmology, Shanghai First People's Hospital, Shanghai Jiao Tong University, Shanghai 200080, China; ^4^Department of Ophthalmology, Shanghai TCM-Integrated Hospital, Shanghai University of TCM, 230 Baoding Road, Shanghai 200082, China

## Abstract

**Purpose:**

This study aimed to evaluate the mechanisms underlying the effects of 1,25-dihydroxyvitamin D (vitamin D_3_) on diabetes-induced retinal vascular damage and retinal vascular endothelial cell apoptosis.

**Methods:**

Diabetic and control rats were randomly assigned to receive vitamin D_3_ or vehicle for 6 months. Additionally, human retinal microvascular endothelial cells (HRMECs) were incubated in normal or high-glucose medium with or without vitamin D_3_. Morphological changes in retinal tissues and retinal vascular permeability were examined, and cellular apoptosis was detected by fluorescence staining. Intracellular reactive oxygen species (ROS) levels were determined using fluorescent probes. Proteins were examined by Western blotting.

**Results:**

Vitamin D_3_ significantly downregulated intracellular ROS and inhibited TRX-interacting protein (TXNIP)/NOD-like receptor family, pyrin domain-containing 3 (NLRP3) inflammasome pathway activation. Additionally, vitamin D_3_ reduced vascular endothelial growth factor (VEGF) expression and the Bax/Bcl-2 ratio. These changes were associated with retinal recovery and with decreases in retinal vascular permeability and retinal capillary cell apoptosis.

**Conclusions:**

Vitamin D_3_ decreases diabetes-induced ROS and exerts protective effects against retinal vascular damage and cell apoptosis in association with inhibition of the ROS/TXNIP/NLRP3 inflammasome pathway. Understanding the mechanisms of action of vitamin D_3_ has important implications for preventing and treating inflammatory-related illnesses such as diabetic retinopathy.

## 1. Introduction

The total number of patients with diabetes worldwide is expected to increase from 171 million in 2000 to 366 million by 2030 and constitutes one of the major threats to global health [1]. Regarding vascular complications of diabetes, 35% of individuals with diabetes have some form of diabetic retinopathy (DR), 7% have proliferative DR (PDR), 7% have diabetic macular edema, and 10% are in vision-threatening stages [2]. Therefore, identifying methods of inhibiting the development of DR has become a focus of investigation.

Vitamin D is a multifunctional hormone, as its activated metabolite, 1,25-dihydroxy vitamin D_3_, has many diverse biological functions in addition to its classical role in calcium and bone homeostasis, including blood pressure control, immunoregulation, apoptosis inhibition, and antiangiogenesis [3, 4]. Moreover, vitamin D_3_ plays an important role in endothelial function and exerts antioxidant and anti-inflammatory effects [5–7]. Recently, several epidemiological and clinical studies demonstrated the association between vitamin D_3_ and DR [8–12]. At the genetic level, several polymorphisms in the vitamin D receptor (VDR) are associated with an increased risk of DR [13–16]. There is accumulating evidence that hypovitaminosis D is closely related to DR. Payne et al. reported that patients with diabetes, especially those with PDR, have lower 25(OH)D levels than those without diabetes [17]. Ahmadieh et al. found that a low serum 25(OH)D level was an independent predictor of DR in patients with diabetes mellitus type 2 (DM2). Further research has indicated that vitamin D prevents the development and progression of DR by inhibiting inflammation and neovascularization in retinal tissues [18]. Specifically, vitamin D might decrease the proliferation of immunocytes and the expression of proinflammatory cytokines and downregulate the transcriptional activity of hypoxia-inducible factor-1 as well as its target genes, such as vascular endothelial growth factor (VEGF). However, Bonakdaran et al. reported in a clinic-based cross-sectional study that decreased serum vitamin D levels were not associated with an increased risk of DR or its risk factors. Thus, the mechanism by which vitamin D_3_ inhibits DR remains to be elucidated.

Chronic inflammation underlies much of the pathology of DR [19]. Many physiologic and molecular abnormalities that are consistent with inflammation are increased in the retinas or vitreous humour of diabetic animals and patients [20, 21]. In particular, this diabetes-induced inflammation is grossly apparent in the retina based on the levels of proinflammatory cytokines, such as tumor necrosis factor-alpha (TNF*α*), interleukin- (IL-) 1-beta (IL-1*β*), VEGF, IL-8, and IL-6 [22–24]. In light of the evidence for the role of IL-1*β* in inflammation in DR, we hypothesized a role for the NOD-like receptor family, pyrin domain-containing 3 (NLRP3) inflammasome in DR pathogenesis. The NLRP3 inflammasome is a multiprotein complex composed of the adaptor protein apoptosis-associated speck-like protein containing a CARD (ASC; also known as PYCARD), the effector cysteine protease 1 (caspase 1), and NLRP3. The NLRP3 inflammasome induces inflammation by mediating the catalytic activation of caspase-1, followed by the cleavage and release of the proinflammatory cytokines IL-1*β* and IL-18 [25, 26]. Consequently, we can infer that the NLRP3/interleukin-1*β* pathway plays a critical role in the development and progression of DR.

Vitamin D_3_ is essential for a vast number of physiologic processes. In particular, vitamin D_3_ may have a suppressive role in the pathogenesis of inflammation and angiogenesis, which are features of DR. Therefore, we hypothesized that vitamin D_3_ protects against the development of DR via its anti-inflammatory and antiangiogenic effects.

## 2. Materials and Methods

All experiments in this study complied with the requirements of the Association for Research in Vision and Ophthalmology statement with regard to the “Use of Animals in Ophthalmic and Vision Research.” All chemicals were of reagent grade and were purchased from Sigma Chemicals (St. Louis, MO, USA) unless stated otherwise.

### 2.1. Animals

Eight-week-old male Sprague-Dawley rats weighing 180–200 g (Shanghai Laboratory Animal Center, Chinese Academy of Sciences) were randomly assigned to receive either 60 mg/kg streptozotocin (STZ) intraperitoneally or citrate buffer alone. Rats were categorized as diabetic when their blood glucose exceeded 16.7 mmol/L at 48 h after STZ administration. Control animals were age matched and given an equal volume of sodium citrate alone instead of STZ. All rats were randomly divided into four groups (*n* = 10 each):

- Normal control group (NC): SD rats given sodium citrate and administered intramuscular injections of tea oil
- Normal control group + vitamin D_3_ (NC + VD): SD rats administered sodium citrate and intramuscular injections of calcitriol (General, Shanghai, China) at a dose of 233.3 U/kg body weight/week dissolved in tea oil for 6 months, starting 4 weeks after diabetes induction
- Type 1 diabetes mellitus group (DM): STZ-induced SD diabetic rats administered intramuscular injections of tea oil
- Type 1 diabetes mellitus group + vitamin D_3_ (DM + VD): STZ-induced SD diabetic rats administered intramuscular injections of calcitriol at a dose of 233.3 U/kg body weight/week dissolved in tea oil for 6 months, starting 4 weeks after the induction of diabetes

Different doses (116.7–466.7 U/kg body weight/week) of vitamin D_3_ were tested in a preliminary experiment. The dose of 233.3 U/kg body weight/week was the minimum effective dose.

### 2.2. Cell Culture and Treatment

Human retinal microvascular endothelial cells (HRMECs) (Cat. number ACBRI 181, Kirkland, WA, USA) were purchased from Cell Systems with CSC complete medium (glucose concentration of 5 mmol/L), and cells at passage 3–7 were used in the following experiment. Confluent cells were switched to serum-free medium for 24 h before treatment. The cells were exposed to normal glucose (5 mmol/L), normal glucose plus vitamin D_3_ (50 nM, D1530 Sigma, high glucose (30 mmol/L), high glucose plus vitamin D_3_ (50 nM), high glucose after transfection with an interference plasmid targeting NLRP3, normal glucose after transfection with an overexpression plasmid expressing NLRP3, and high glucose plus the reactive oxygen species (ROS) scavenger N-acetylcysteine (NAC; 10 mmol/L). Each group was incubated for 72 h. In addition, we also added mannitol to the normal glucose medium for osmotic compensation.

### 2.3. Plasmid DNA and Transfections

**Figure 1:**
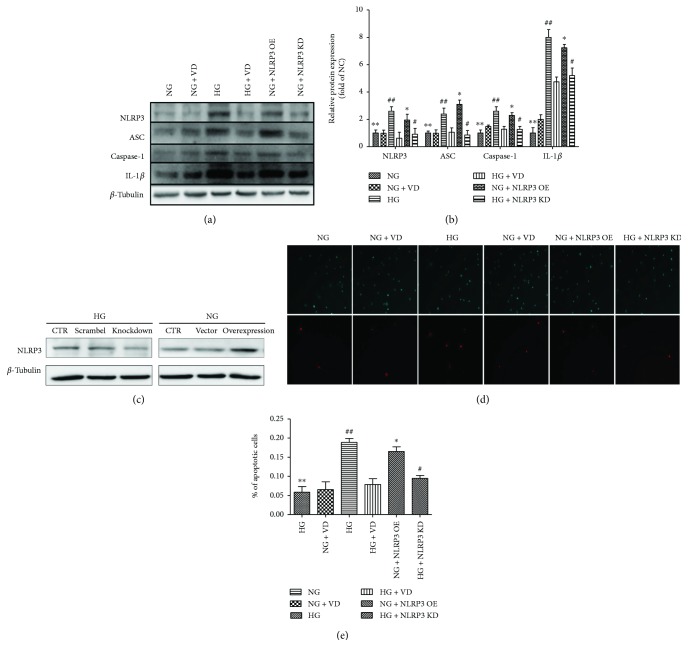
Vitamin D_3_ inhibits the high-glucose-induced death of HRMECs by inhibiting expression of proteins in the NLRP3 inflammasome pathway. (a) Western blot analysis of proteins in the NLRP3 inflammasome pathway in the NG, NG + VD, HG, HG + VD, and NG + NLRP3-overexpressing and HG + NLRP3-knockdown groups. (b) Quantitative analysis of the bands. (c) Transfection of HRMECs with plasmid DNA (CTR: control group; scramble: scrambled shRNA control group; vector: empty vector control group). (d) Co-staining of Hoechst33238 and propidium iodide was performed to assess apoptosis of HRMECs. (e) Quantification of apoptotic cells. The results are expressed as the mean percentage of apoptotic cells ± SD. *n* = 6 (∗∗: NG versus HG, *P* < 0.05; ##: HG versus HG + VD, *P* < 0.05; ∗: NG + NLRP3 Op versus NG, *P* < 0.05; #: HG + NLRP3 KD versus HG, *P* < 0.05).

Transient transfection was carried out using Lipofectamine 2000 according to the manufacturer's protocol (Invitrogen, Grand Island, NY, USA). Briefly, HRMECs were seeded at a density of 10^6^/60 mm plate overnight and then cultured in serum-free culture medium containing 4 *μ*g pDNA and 10 *μ*L Lipofectamine 2000 for 6 h. Transfected cells were then switched to normal medium for 12 h before subsequent experiments. The target short hairpin (sh) RNA sequences were 5′-GGAGAGACCTTTATGAGAAAG-3′ (Dharmacon) for NLRP3 knockdown. The plasmids encoding full-length NLRP3 were provided by Dr. Haibin Chen (Department of Endocrinology and Metabolism, the Sixth People's Hospital of Shanghai Affiliated to Shanghai Jiao Tong University, Shanghai, China). Both scrambled shRNA control (for knockdown) and empty vector (for overexpression) were used for appropriate controls (Figure 1(c)).

### 2.4. Histopathological Evaluation

In the article of Li et al. [27], authors demonstrated that rosiglitazone attenuated diabetes-induced rat retinal structural abnormality by histopathological evaluation. We followed the methods of Li et al. [27]. Histopathological evaluation was examined by a light microscope (Leica, Heidelberg, Germany). Coloured micrographs were obtained at ×400 magnification. Measurements (×400) were made 0.5 mm dorsal and 0.5 mm ventral to the optic disc. The thicknesses of the total retina (between the inner limiting membrane and pigment epithelium), inner plexiform layer (IPL), and inner nuclear layer (INL) and the combined thickness of the outer plexiform and outer nuclear layers (pooled as the outer retinal layers, ORL) were measured. The number of cells in the ganglion cell layer (GCL) was calculated based on the linear cell density (cells per 100 *μ*m). Three measurements were made at adjacent locations in each hemisphere, and the nine measurements were averaged. The mean of the three eyes was recorded as the representative value for each group. The results of the three groups were subjected to statistical analysis.

### 2.5. Apoptosis of Retinal Cells

The Dead End Fluorometric TUNEL system (Roche TUNEL kit; Roche Applied Science, Burgess Hill, UK) was used as described by the manufacturer. Briefly, paraffin sections were prepared as described by the manufacturer and subjected to TUNEL analysis after incubation with proteinase K; Hoechst counterstaining was then performed. Fluorescence images were acquired using a fluorescence microscope (Olympus, Tokyo, Japan), and images were obtained using Zeiss software version 4.6. Apoptotic cells within the retina fluoresced red.

### 2.6. Measurement of Retinal Vascular Permeability

Retinal vascular permeability was analysed by measuring Evans blue-albumin complex leakage from retinal vessels, as described previously [28]. Briefly, Evans blue dye was dissolved in normal saline (45 mg/mL). Then, under deep anaesthesia, the dye (45 mg/kg) was injected into the jugular vein of each rat. Blood (200 *μ*L) was withdrawn from the iliac artery 2 min after Evans blue injection and then every 30 min for up to 120 min. After the dye had circulated for 120 min, the chest cavity was opened, and the left ventricle was cannulated. Each rat was perfused with 0.05 M citrate buffer, pH 3.5 (37°C), for 2 min at 66 mL/min to clear the dye. Immediately after perfusion, the eyes were enucleated, and the retinas were carefully dissected under an operating microscope. Evans blue in the retina and blood samples was detected as described previously [29]. Retinal vascular permeability was calculated using the following equation, and the results were expressed as *μ*L plasma/g retina dry weight/h: retinal vascular permeability = Evans blue (*μ*g)/retina dry weight (g)/time-averaged Evans blue concentration (*μ*g)/plasma (*μ*L) × circulation time (h).

### 2.7. Assessment of HRMEC Apoptosis Using Propidium Iodide (PI)/Hoechst

To assess cellular apoptosis, HRMECs were stained with Hoechst and PI dyes. Blue fluorescent Hoechst 33342 binds to chromatin and stains the condensed chromatin of apoptotic cells more brightly than that of normal cells, whereas red fluorescent PI permeates late-stage apoptotic and dead cells. HRMECs were stained with 10 *μ*g/mL PI and 10 *μ*g/mL Hoechst for 15 min at 37°C in the dark and then visualized and photographed using a fluorescence microscope (Olympus, Tokyo, Japan) to allow the number of apoptotic cells among 500 total cells to be counted. The percentage of red (apoptotic; R) cells versus bright blue (viable; BB) cells was determined as follows: % apoptotic cells = (R + BB)/500.

### 2.8. Measurement of ROS Production

Intracellular ROS levels were determined using the oxidation-sensitive fluorescent probe 2′,7′-dichlorofluorescein diacetate (H2DCF-DA). Intracellular ROS oxidize this probe into the highly fluorescent compound DCF. Cells were incubated with 10 *μ*M H2DCF-DA in medium at 37°C for 30 min, and images were acquired using a fluorescence microscope and quantified using ImageJ (National Institutes of Health, Bethesda, MD, USA).

### 2.9. Western Blotting

Total protein samples were separated using 10–12% SDS-polyacrylamide gels and then incubated with the following primary antibodies: NLRP3 (goat polyclonal; Santa Cruz Biotechnology), apoptosis-associated speck-like protein containing a caspase recruitment domain (ASC, rabbit monoclonal; Adipogen International Inc., San Diego, CA, USA), caspase-1 (rabbit polyclonal; Santa Cruz Biotechnology), IL-1*β* (rabbit polyclonal; Abcam, Cambridge, UK), TXNIP (goat monoclonal; MBL International), vascular endothelial growth factor (VEGF, goat polyclonal; Santa Cruz Biotechnology), BAX (Cell Signaling Technology, Beverly, MA, USA), Bcl-2 (Cell Signaling Technology), and *β*-tubulin (rabbit polyclonal; Bioworld Technology Inc., St. Louis Park, MN, USA). The blots were washed with Tris-buffered saline containing Tween 20, and signals were developed using enhanced chemiluminescence (Western Chemiluminescent HRP Substrate; EMD Millipore, Billerica, MA, USA).

### 2.10. Statistical Analysis

Results are expressed as mean ± standard deviation (SD). Group means were compared by one-way analysis of variance using the GraphPad Prism software (ver. 4.0; GraphPad, San Diego, CA) and the SPSS software (ver. 17.0 for Windows; SPSS Inc., Chicago, IL). In all comparisons, a value of *P* < 0.05 was considered to indicate a statistical significance.

## 3. Results

### 3.1. Vitamin D_3_ Inhibits the Decrease in GCL Cell Numbers and Retinal Thickness in Diabetic Rats

**Figure 2:**
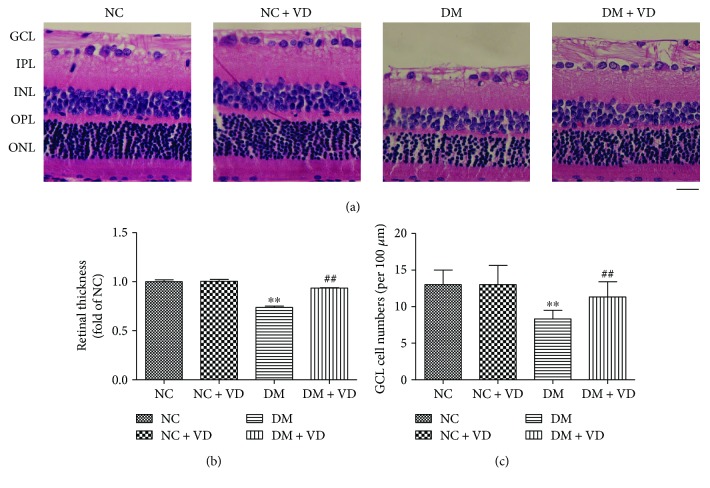
(a) Representative images of retinas at month 6. (b-c) The retinal thickness and cell numbers in the GCL (cells per 100 *μ*m). At month 6, the GCL and IPL were obvious and well organized in the NC group. The retinal thickness in diabetic rats was reduced and the number of GCL cells decreased (DM), whereas in diabetic rats treated with vitamin D_3_, the retinas were more normal in structure and thicker than those in diabetic rats (NC: normal control group; NC + VD: normal control group + vitamin D_3_; DM: diabetes mellitus group; IPL: inner plexiform layer; GCL: ganglion cell layer). Data represent mean ± SD (*n* = 10). ^∗∗^DM versus NC, *P* < 0.05; ^##^DM + VD versus DM, *P* < 0.05. Data are expressed as the mean ± standard deviation. Scale bar, 30 *μ*m.

The thicknesses of the total retina, IPL, INL, and ORL and the density of GCL cells in the diabetes mellitus group were significantly lower than those in the NC group (*P* < 0.05, Table 1). The cells in each layer of retinal tissue were shaped more abnormally, arranged in a more disorderly fashion and of a lower density (cells per 100 *μ*m) in the DM group than the NC group. However, the retinal structure and cell density of diabetic rats were similar to those in the NC group after treatment with vitamin D_3_ (Figure 2(a)). Likewise, measurements of retinal thicknesses and GCL cell numbers showed a significant increase in the DM + VD group compared with the DM group, which confirmed the protective effect of vitamin D_3_ on the decrease in GCL cell numbers and retinal thickness in diabetic rats (Table 1, Figures 2(b) and 2(c)).

### 3.2. Vitamin D_3_ Inhibits Retinal Cell Apoptosis in Diabetic Rats by Decreasing the Bax/Bcl-2 Ratio

**Figure 3:**
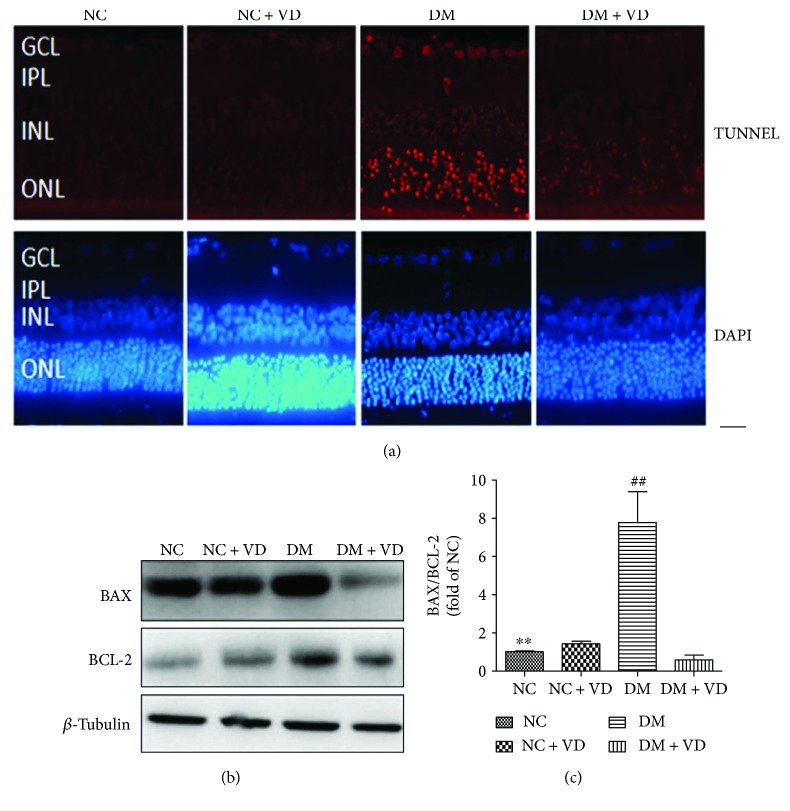
Protective effect of vitamin D_3_ against apoptosis of retinal cells at month 6. (a) No TUNEL-positive cells were seen in the NC or NC + VD groups; abundant red fluorescent nuclei were observed in the DM group. Sparse red fluorescent nuclei were found in the DM + VD group. GCL: ganglion cell layer; INL: inner nuclear layer; ONL: outer nuclear layer. (b) Western blot of retinal extracts using anti-Bcl-2 and anti-Bax antibodies; *β*-tubulin was used as the loading control. (c) Bax/Bcl-2 ratios (data represent mean ± SD (*n* = 10) ∗∗: NC versus DM, *P* < 0.05, ##: DM versus DM + VD, *P* < 0.05. NC: normal control group; NC + VD: normal control group + vitamin D_3_; DM: diabetes mellitus group; DM + VD: diabetes mellitus group + vitamin D_3_. Scale bar, 30 *μ*m.

To examine retinal cell death, TUNEL staining was performed at month 6 (Figure 3(a)). In the NC and NC + VD groups, the retinal cell nuclei were negative for TUNEL staining, showing no red fluorescence. There were abundant red fluorescent retinal cell nuclei, indicating TUNEL-positive cells, within the inner nuclear, outer nuclear, and ganglion cells in the diabetic rats. In contrast, in the DM + VD group, only sparse red fluorescent nuclei were found in rat retinas by TUNEL staining.

The decreased apoptosis in diabetic rats following vitamin D_3_ treatment could be due to changes in Bcl-2 family proteins, which are well-known inducers and integrators of survival and death signals. Therefore, we next determined whether hyperglycaemia affected the levels of the proapoptotic molecule Bax and the antiapoptotic molecule Bcl-2 in diabetic rats using immunoblotting. The Bax/Bcl-2 ratio was upregulated almost eightfold in retinal extracts from diabetic rats and was downregulated by 93.5% after treatment with vitamin D_3_ in diabetic rats' retinal extracts.

### 3.3. Vitamin D_3_ Attenuates the Increase in Blood-Retinal Barrier (BRB) Breakdown in Diabetic Rats by Inhibiting VEGF Expression and the TXNIP/NLRP3 Pathway

**Figure 4:**
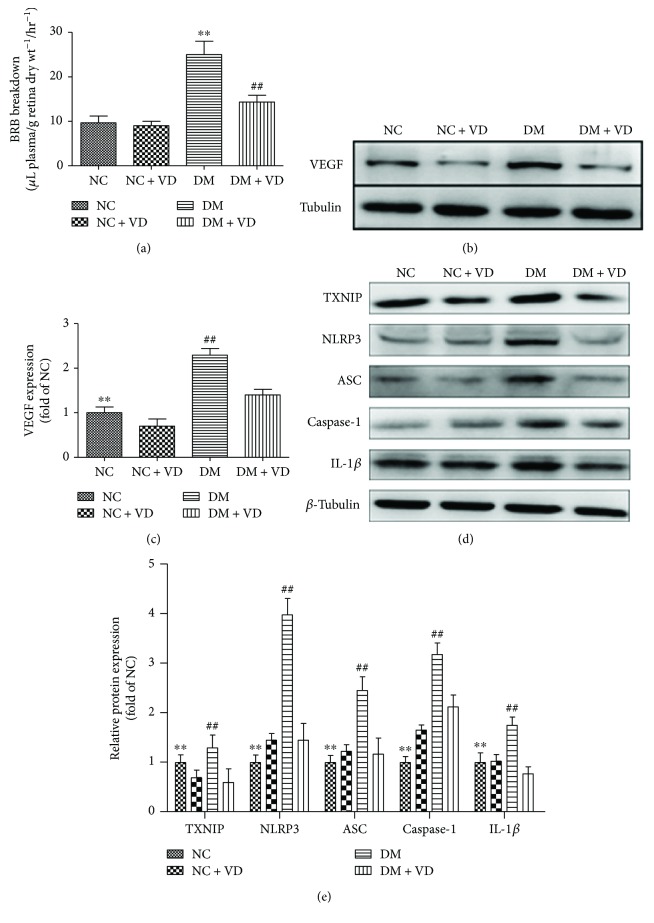
Vitamin D_3_ attenuates the elevated BRB breakdown in diabetic rats by inhibiting VEGF expression and the TXNIP/NLRP3 pathway. (a) Retinal vascular permeability was quantified by detecting the retinal Evans blue leakage (data are the means ± SD, *n* = 10∗∗: DM versus NC, *P* < 0.05 and ##: DM + VD versus DM, *P* < 0.05). (b, c) Retinal VEGF protein expression was inhibited by vitamin D_3_ (data are the means ± SD, *n* = 10∗∗: DM versus NC, *P* < 0.05 and ##: DM + VD versus DM, *P* < 0.05). (d, e) Quantitative Western blot analyses of TXNIP and the NLRP3 inflammasome in whole retinal extracts. Expression was normalized to that of *β*-tubulin. Data are the means ± SD, *n* = 10 (∗∗: NC versus DM, *P* < 0.05; ##: DM + VD versus DM, *P* < 0.05).

Retinal vascular permeability was quantified by detecting retinal Evans blue leakage. As shown in Figure 4(a), BRB permeability was significantly increased in diabetic rat retinas (DM: 25 ± 3 L plasma/g retina dry weight/h) relative to that in control animals (NC: 9 ± 1 L plasma/g retina dry weight/h; *P* < 0.05 versus DM). In diabetic rats treated with vitamin D_3_, there was a significant decrease in BRB permeability (DM + VD: 14 ± 1 L plasma/g retina dry weight/h; *P* < 0.05 versus DM) compared with diabetic animals. Furthermore, VEGF expression and the TXNIP/NLRP3 pathway in rat retinas showed similar trends (Figures 4(c) and 4(e)). The diabetes-induced upregulation of VEGF and proteins in the TXNIP/NLRP3 pathway was prevented by vitamin D_3_ treatment.

### 3.4. Vitamin D_3_ Inhibits Hyperglycaemia-Induced Elevation of TXNIP by Reducing ROS Production in HRMECs

**Figure 5:**
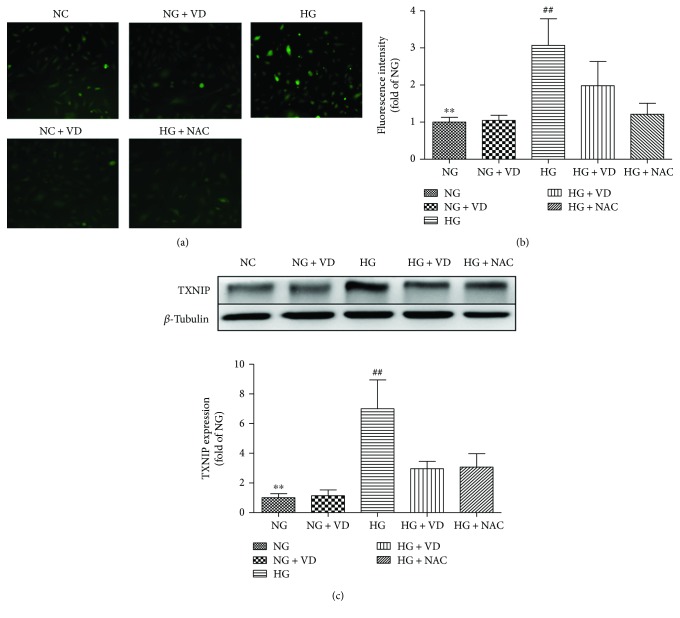
Vitamin D_3_ inhibits glucose-induced ROS production in HRMECs. (a) To measure ROS production, cells were labelled with H2DCF-DA. The figures show representative data from three independent experiments. (b) Quantitative analysis of the measured fluorescence intensity was performed. Values are the means ± SD of three independent experiments (*n* = 6, ∗∗: HG versus NG, *P* < 0.05, ##: HG + VD and HG + NAC versus HG, *P* < 0.05). (c) Western blot and quantitative analyses of TXNIP from whole retinal extracts. Expression was normalized to that of *β*-tubulin. Data are the means ± SD, *n* = 6 (∗∗: HG versus NG, *P* < 0.05, ##: HG + VD and HG + NAC versus HG, *P* < 0.05).

To assess the mechanism underlying the inhibitory effect of vitamin D_3_ on the TXNIP/NLRP3 pathway, an in vitro assay was performed. HRMECs were incubated in low- or high-glucose medium together with 50 nM vitamin D_3_ or 10 mM NAC for 48 h. As shown in Figures 5(a)–5(c), high glucose enhanced ROS generation and increased expression of TXNIP (*P* < 0.05), whereas these changes were significantly inhibited by 50 nM vitamin D_3_, which was comparable to the effect induced by treatment with NAC, an ROS scavenger (*P* < 0.05). The cells were treated with mannitol to eliminate the influence of osmolarity; the results showed that mannitol had no effect (data not shown).

### 3.5. Vitamin D_3_ Inhibits High-Glucose-Induced Death of HRMECs by Inhibiting the Expression of the NLRP3 Inflammasome Pathway

To confirm the mechanism underlying the effect of vitamin D_3_ on HRMEC apoptosis, we performed Western blotting and an apoptosis assay (Figures 1(a) and 1(d)). HRMECs were treated with vitamin D_3_ or plasmid DNA in the presence of various glucose concentrations. When HRMECs were incubated in high-glucose medium for 48 h, the levels of NLRP3 inflammasome pathway proteins increased dramatically, and the rate of apoptosis increased by 4.2-fold (*P* < 0.05). In contrast, the presence of vitamin D_3_ in high-glucose medium prevented the increase in the levels of NLRP3 inflammasome pathway proteins and decreased apoptosis by 59.7% (*P* < 0.05). Furthermore, we used plasmid DNA to transfect HRMECs, of which the transfection effects were significant (Figure 1(c)). Consistently, in HRMECs, the levels of NLRP3 inflammasome pathway proteins increased by overexpressing NLRP3 but decreased by knocking down NLRP3. Meanwhile, the rate of apoptosis was regulated by NLRP3 expression. More specifically, the apoptosis rate was significantly reduced by NLRP3 silencing and increased by NLRP3 overexpressing compared with the nontransfected group.

## 4. Discussion

During the past decade, theories on the functions of vitamin D_3_ have been proposed. In addition to its well-established role in calcium and bone homeostasis, vitamin D_3_ has been shown to exert autocrine or paracrine immunomodulatory effects [30]. Much epidemiological research has shown that vitamin D_3_ deficiency is associated with a large number of autoimmune and inflammatory diseases, such as rheumatoid arthritis, lupus, inflammatory bowel disease, transplant rejection, cardiovascular disease, infections, and diabetes [6]. Thus, an understanding of the underlying anti-inflammatory mechanisms of vitamin D_3_ may extend its clinical applications.

Recently, the relationship between vitamin D_3_ deficiency and DR has become a focus of study [8, 9]. Our study found for the first time that vitamin D_3_ has a protective effect on DR. Vitamin D_3_ markedly reduced retinal injury associated with diabetes, as demonstrated by reduced histological damage and permeability recovery and apoptosis of retinal cells. We also confirmed that the TXNIP/NLRP3 pathway was activated due to increased ROS production induced by high glucose. Vitamin D_3_ exerted its protective effect by decreasing the level of ROS production, thus downregulating TXNIP expression and blocking the activation of NLRP3. Eventually, this could lead to the downregulation of IL-1*β*, which might mitigate retinal inflammation and protect the retina against hyperglycaemia-induced dysfunction.

In an animal study, we found that NLRP3 activation was significantly correlated with damage to retinal cells and vascular permeability, whereas vitamin D_3_ downregulated NLRP3, which subsequently reduced the rate of apoptosis and reduced vascular permeability in the retinas of diabetic rats. Furthermore, we found that the Bax/Bcl-2 protein ratio decreased after high-glucose-treated HRMECs were treated with vitamin D_3_. The ratio of proapoptotic Bax to antiapoptotic Bcl-2 in the retina reflects the level of retinal cell apoptosis in diabetic rats [31]. A previous study found that IL-1*β* induced retinal mitochondrial dysfunction, mitochondrial DNA damage, and cell apoptosis [32]. Bax is a marker of mitochondrial stress associated with vascular diabetes complications [33]. Hence, vitamin D_3_ likely reduced the expression of proapoptotic proteins by inhibiting NLRP3-mediated IL-1*β* secretion. Undoubtedly, VEGF plays a key role in retinal vascular permeability. It was reported that TXNIP increased the expression of VEGF in the diabetic rat retina [34]. In our study, TXNIP exhibited the same trend as VEGF, the levels of which were increased under high-glucose conditions and reversed by vitamin D_3_ treatment. These findings indicate that the protective effect of vitamin D_3_ on DR is associated with inhibition of the TXNIP/NLRP3 pathway, which may serve as an effective marker of the progression, prevention, and treatment of DR.

To explore the mechanism underlying the protective effect of vitamin D_3_ on DR, we performed experiments in vitro. The elevated levels of NLRP3 inflammasome proteins induced by high glucose resulted in high expression of IL-1*β*, which led to a cellular inflammatory response and death. Overexpression of NLRP3 in the NG group produced similar findings. We also noticed that vitamin D_3_ reversed the high-glucose-induced increase in NLRP3 inflammasome protein levels and apoptosis, an effect that was equivalent to the knockdown of NLRP3. These findings suggest that vitamin D_3_ protects HRMECs by inhibiting the increase in NLRP3 inflammasome protein levels and decreasing IL-1*β* secretion.

In our previous studies, we verified that high glucose induces ROS production [35, 36]. Hyperglycaemia-induced ROS production is thought to be a common upstream event in all four classic pathogenetic pathways of diabetic microangiopathy [37–39]. Additionally, two previous studies confirmed that ROS generation is crucial for NLRP3 activation [40, 41]. All NLRP3 agonists trigger the production of ROS, which leads to activation of the NLRP3 inflammasome via the ROS-sensitive TXNIP protein [42]. TXNIP has been reported to be induced by high glucose, and ROS trigger the dissociation of TXNIP from thioredoxin, which alters the function of TXNIP from a thioredoxin repressor to an NLRP3 inflammasome activator. We speculate that vitamin D_3_ inhibits NLRP3 by reducing the expression of TXNIP and ROS production. We determined ROS levels and TXNIP expression in HRMECs incubated in the presence of high glucose and NAC. Consistently, our results showed that vitamin D_3_ inhibits the expression of TXNIP by degrading ROS in high-glucose-induced HRMECs.

In conclusion, using in vitro and in vivo studies, we demonstrated that vitamin D_3_ can protect the normal retinal structure, alleviate retinal vascular permeability, and inhibit the apoptosis of retinal cells in diabetic rats. We also provided evidence that this protective effect was caused by inhibition of the TXNIP/NLRP3 pathway as a result of reduced ROS production in high-glucose-induced retinal microvascular endothelial cells. Thus, vitamin D_3_ possesses considerable potential for the treatment of DR. A long-term prospective study with a large number of samples is needed to verify the clinical effect of vitamin D_3_ in DR.

## Figures and Tables

**Table 1:** Thicknesses of the retinal layers and ganglion cell layer (GCL) and cell counts at month 6.

	Thickness (*μ*m)				
	Total retina	IPL	INL	ORL	GCL cell numbers (per 100 *μ*m)
NC (*n* = 10)	243.1 ± 3.2	74.3 ± 2.0	39.2 ± 2.1	73 ± 3.5	13.0 ± 1.5
NC + VD (*n* = 10)	245.5 ± 4.1	79.6 ± 2.8	41.2 ± 2.7	76 ± 2.9	13.0 ± 2.1
DM (*n* = 10)	176.2 ± 3.7^∗^	54.9 ± 3.4^∗^	37.3 ± 3.2^∗^	65 ± 3.5^∗^	8.3 ± 0.8^∗^
DM + VD (*n* = 10)	229.8 ± 2.7^#^	68.7 ± 3.2^#^	38.2 ± 3.1^#^	69 ± 3.0^#^	11.3 ± 1.3^#^

NC: normal control group; NC + VD: normal control group + vitamin D_3_; DM: diabetes mellitus group; DM + VD: diabetes mellitus group + vitamin D_3_; IPL: inner plexiform layer; INL: inner nuclear layer; ORL: outer retinal layers; GCL: ganglion cell layer. Data represent mean ± SD (*n* = 10). ^∗^NC versus DM, *P* < 0.05; ^#^DM + VD versus DM, *P* < 0.05. Data are expressed as the mean ± standard deviation.

## References

[1] Wild S., Roglic G., Green A., Sicree R., King H. (2004). Global prevalence of diabetes: estimates for the year 2000 and projections for 2030. *Diabetes Care*.

[2] Yau J. W. Y., Rogers S. L., Kawasaki R. (2012). Global prevalence and major risk factors of diabetic retinopathy. *Diabetes Care*.

[3] Plum L. A., DeLuca H. F. (2010). Vitamin D, disease and therapeutic opportunities. *Nature Reviews Drug Discovery*.

[4] Prietl B., Treiber G., Pieber T. R., Amrein K. (2013). Vitamin D and immune function. *Nutrients*.

[5] Chagas C. E. A., Borges M. C., Martini L. A., Rogero M. M. (2012). Focus on vitamin D, inflammation and type 2 diabetes. *Nutrients*.

[6] Guillot X., Semerano L., Saidenberg-Kermanac’h N., Falgarone G., Boissier M.-C. (2010). Vitamin D and inflammation. *Joint, Bone, Spine*.

[7] Tarcin O., Yavuz D. G., Ozben B. (2009). Effect of vitamin D deficiency and replacement on endothelial function in asymptomatic subjects. *The Journal of Clinical Endocrinology & Metabolism*.

[8] Aksoy H., Akçay F., Kurtul N., Baykal O., Avci B. (2000). Serum 1,25 dihydroxy vitamin D (1,25(OH)_2_D_3_), 25 hydroxy vitamin D (25(OH)D) and parathormone levels in diabetic retinopathy. *Clinical Biochemistry*.

[9] Kaur H., Donaghue K. C., Chan A. K. (2011). Vitamin D deficiency is associated with retinopathy in children and adolescents with type 1 diabetes. *Diabetes Care*.

[10] Patrick P. A., Visintainer P. F., Shi Q., Weiss I. A., Brand D. A. (2012). Vitamin D and retinopathy in adults with diabetes mellitus. *Archives of Ophthalmology*.

[11] Longo-Mbenza B., Mvitu Muaka M., Masamba W. (2014). Retinopathy in non diabetics, diabetic retinopathy and oxidative stress: a new phenotype in Central Africa?. *International Journal of Ophthalmology*.

[12] Shimo N., Yasuda T., Kaneto H. (2014). Vitamin D deficiency is significantly associated with retinopathy in young Japanese type 1 diabetic patients. *Diabetes Research & Clinical Practice*.

[13] Taverna M. J., Selam J.-L., Slama G. (2005). Association between a protein polymorphism in the start codon of the vitamin D receptor gene and severe diabetic retinopathy in C-peptide-negative type 1 diabetes. *The Journal of Clinical Endocrinology & Metabolism*.

[14] van Etten E., Verlinden L., Giulietti A. (2007). The vitamin D receptor gene *Fok*I polymorphism: functional impact on the immune system. *European Journal of Immunology*.

[15] Taverna M. J., Sola A., Guyot-Argenton C. (2002). Taq I polymorphism of the vitamin D receptor and risk of severe diabetic retinopathy. *Diabetologia*.

[16] Zhong X., Du Y., Lei Y., Liu N., Guo Y., Pan T. (2015). Effects of vitamin D receptor gene polymorphism and clinical characteristics on risk of diabetic retinopathy in Han Chinese type 2 diabetes patients. *Gene*.

[17] Payne J., Ray R., Watson D. (2012). Vitamin D insufficiency in diabetic retinopathy. *Endocrine Practice*.

[18] Ren Z., Li W., Zhao Q., Ma L., Zhu J. (2012). The impact of 1, 25-dihydroxy vitamin D_3_ on the expressions of vascular endothelial growth factor and transforming growth factor-*β*_1_ in the retinas of rats with diabetes. *Diabetes Research and Clinical Practice*.

[19] Adamis A. P. (2002). Is diabetic retinopathy an inflammatory disease?. *British Journal of Ophthalmology*.

[20] Kern T. S. (2007). Contributions of inflammatory processes to the development of the early stages of diabetic retinopathy. *Experimental Diabetes Research*.

[21] Tang J., Kern T. S. (2011). Inflammation in diabetic retinopathy. *Progress in Retinal and Eye Research*.

[22] Kocak N., Alacacioglu I., Kaynak S. (2010). Comparison of vitreous and plasma levels of vascular endothelial growth factor, interleukin-6 and hepatocyte growth factor in diabetic and non-diabetic retinal detachment cases. *Annals of Ophthalmology*.

[23] Vincent J. A., Mohr S. (2007). Inhibition of caspase-1/interleukin-1*β* signaling prevents degeneration of retinal capillaries in diabetes and galactosemia. *Diabetes*.

[24] Behl Y., Krothapalli P., Desta T., DiPiazza A., Roy S., Graves D. T. (2008). Diabetes-enhanced tumor necrosis factor-*α* production promotes apoptosis and the loss of retinal microvascular cells in type 1 and type 2 models of diabetic retinopathy. *The American Journal of Pathology*.

[25] Ting J. P.-Y., Willingham S. B., Bergstralh D. T. (2008). NLRs at the intersection of cell death and immunity. *Nature Reviews Immunology*.

[26] Schroder K., Tschopp J. (2010). The inflammasomes. *Cell*.

[27] Li P., Xu X., Zheng Z., Zhu B., Shi Y., Liu K. (2011). Protective effects of rosiglitazone on retinal neuronal damage in diabetic rats. *Current Eye Research*.

[28] Leal E. C., Martins J., Voabil P. (2010). Calcium dobesilate inhibits the alterations in tight junction proteins and leukocyte adhesion to retinal endothelial cells induced by diabetes. *Diabetes*.

[29] Qaum T., Xu Q., Joussen A. M. (2001). VEGF-initiated blood-retinal barrier breakdown in early diabetes. *Investigative Ophthalmology & Visual Science*.

[30] Hewison M. (2012). Vitamin D and the immune system: new perspectives on an old theme. *Rheumatic Diseases Clinics of North America*.

[31] Gao X.-Y., Kuang H.-Y., Zou W., Liu X. M., Lin H. B., Yang Y. (2009). The timing of re-institution of good blood glucose control affects apoptosis and expression of Bax and Bcl-2 in the retina of diabetic rats. *Molecular Biology Reports*.

[32] Kowluru R. A., Mohammad G., Santos J. M., Tewari S., Zhong Q. (2011). Interleukin-1*β* and mitochondria damage, and the development of diabetic retinopathy. *Journal of Ocular Biology, Diseases, and Informatics*.

[33] Podestà F., Romeo G., Liu W.-H. (2000). Bax is increased in the retina of diabetic subjects and is associated with pericyte apoptosis *in vivo* and *in vitro*. *The American Journal of Pathology*.

[34] Devi T. S., Lee I., Hüttemann M., Kumar A., Nantwi K. D., Singh L. P. (2012). TXNIP links innate host defense mechanisms to oxidative stress and inflammation in retinal Muller glia under chronic hyperglycemia: implications for diabetic retinopathy. *Experimental Diabetes Research*.

[35] Zheng Z., Chen H., Ke G. (2009). Protective effect of perindopril on diabetic retinopathy is associated with decreased vascular endothelial growth factor–to–pigment epithelium–derived factor ratio involvement of a mitochondria–reactive oxygen species pathway. *Diabetes*.

[36] Zheng Z., Chen H., Wang H. (2010). Improvement of retinal vascular injury in diabetic rats by statins is associated with the inhibition of mitochondrial reactive oxygen species pathway mediated by peroxisome proliferator–activated receptor *γ* coactivator 1*α*. *Diabetes*.

[37] Forbes J. M., Coughlan M. T., Cooper M. E. (2008). Oxidative stress as a major culprit in kidney disease in diabetes. *Diabetes*.

[38] Brownlee M. (2001). Biochemistry and molecular cell biology of diabetic complications. *Nature*.

[39] Brownlee M. (2005). The pathobiology of diabetic complications: a unifying mechanism. *Diabetes*.

[40] Tschopp J., Schroder K. (2010). NLRP3 inflammasome activation: the convergence of multiple signalling pathways on ROS production?. *Nature Reviews Immunology*.

[41] Lawlor K. E., Vince J. E. (2014). Ambiguities in NLRP3 inflammasome regulation: is there a role for mitochondria?. *Biochimica et Biophysica Acta (BBA)-General Subjects*.

[42] Schroder K., Zhou R., Tschopp J. (2010). The NLRP3 inflammasome: a sensor for metabolic danger?. *Science*.

